# Cerebrospinal fluid level of neurofilament light chain is associated with increased disease activity in neuro-Behçet’s disease

**DOI:** 10.55730/1300-0144.5432

**Published:** 2022-04-10

**Authors:** Zerrin KARAASLAN, Elif ŞANLI, Özlem TİMİRCİ-KAHRAMAN, Vuslat YILMAZ, Ece AKBAYIR, Gizem KORAL, Sema İÇÖZ, Tuncay GÜNDÜZ, Cem İsmail KÜÇÜKALİ, Murat KÜRTÜNCÜ, Erdem TÜZÜN

**Affiliations:** 1 Department of Neuroscience, Aziz Sancar Institute of Experimental Medicine, İstanbul University, İstanbul, Turkey; 2 Institute of Graduate Studies in Health Sciences, İstanbul University, İstanbul, Turkey; 3 Department of Molecular Medicine, Aziz Sancar Institute of Experimental Medicine, İstanbul University, İstanbul, Turkey; 4 Department of Neurology, İstanbul Faculty of Medicine, İstanbul University, İstanbul, Turkey

**Keywords:** Neurofilament light, hoxB3, ykl-40, neuro-Behçet, prognosis

## Abstract

**Background/aim:**

Clinical exacerbations characterized with neurological symptoms are observed in around 10% of Behçet’s disease (BD) patients and may culminate in severe disability. Although certain immunological factors have been associated with disease activity in neuro-Behçet’s disease (NBD), biomarkers for monitoring the clinical outcome of NBD have not been properly investigated.

**Materials and methods:**

Levels of neurofilament light chain (NFL), homeobox protein Hox-B3 (HoxB3), and YKL-40 were measured in cerebrospinal fluid (CSF) samples of 23 parenchymal (n = 16) and nonparenchymal (n = 7) NBD patients obtained during NBD attacks by ELISA. Parameters of clinical progression and outcome were assessed for an average follow-up period of 3.9 ± 1.3 years.

**Results:**

Parenchymal NBD patients showed elevated CSF levels of NFL, HoxB3, and YKL-40 as compared to nonparenchymal patients. NBD patients showing an increase in modified Rankin score (mRS) values during follow-up had significantly higher CSF NFL levels. Patients with relatively lower CSF NFL levels (<1000 ng/L) did not develop attacks or cognitive impairment interfering with daily life activities during follow-up. NFL levels correlated with disease duration and mRS at the last follow-up visit, while HoxB3 levels correlated with a number of attacks during follow-up.

**Conclusion:**

CSF level of NFL appears to predict the prospective somatic and cognitive disability in NBD patients and may thus be potentially used as a biomarker of clinical outcome in this disease.

## 1. Introduction

Behçet’s disease (BD) is a multisystem inflammatory disorder characterized by recurrent oral aphthae, genital ulcerations, and uveitis [1]. Central nervous system (CNS) involvement, which is named neuro-Behçet’s disease (NBD) occurs in around 10% of BD patients and manifests in parenchymal and vascular forms. While, in vascular form, thrombosis of the dural sinus and cortical veins are most often encountered, in the parenchymal form, CNS lesions are found in the brainstem, basal ganglia, and diencephalon. Innate immunity is most profoundly activated in BD leading to CNS lesions exhibiting infiltrating macrophages, neutrophils, and activated glial cells [2,3].

NBD often presents with attack and remission periods and a significant accumulation of disability can be observed in some patients after a few attacks occurring in a short time frame [4,5]. Cerebrospinal fluid (CSF) levels of IL-6 and increased CSF cell count are the most consistently reported prognostic biomarkers [6]. IL-6 has also been shown to decrease CSF in parallel to favorable treatment response to tocilizumab [7]. Although CSF levels of IL-6, IL-37, and BAFF have been found to be correlated with disease activity in cross-sectional studies, the significance of these mediators as disease monitoring biomarkers has not been evaluated in longitudinal follow-up studies [8–10]. Thus, biomarkers that predict clinical evolution and long-term outcome of NBD are sorely needed for improved treatment decisions.

In this context, neurofilament light chain (NFL) and chitinase-3-like protein 1, which is also known as YKL-40, have stood out as potential prognostic and monitoring biomarkers in several neuroimmunological and neurodegenerative disorders [11–13]. NFL is a neuronal cytoplasmic protein, which is found in axons and rises in CSF in proportion to the degree of axonal damage. NFL appears to be not only a biomarker of axonal damage but also cognitive impairment in a variety of disorders including multiple sclerosis (MS) and amyotrophic lateral sclerosis [12]. Several studies have shown that CSF levels of YKL-40, which is likely a marker of glial activity [14], effectively discriminate different prognostic subgroups of MS and predict the progression of disability [13,15]. Notably, an association between serum levels of YKL-40 and disease activity has been demonstrated in BD patients [16–18]. Based on our proteomics and ELISA studies, we have recently identified that CSF level of homeobox protein Hox-B3 (HoxB3) may be used to predict conversion from clinically isolated syndrome to MS alone or in combination with YKL-40 [19,20]. Although the exact function of HoxB3 in the CNS is not well understood, its growth regulatory action and high expression in lymphocytes and leukemia cells suggest that HoxB3 might be a marker of immune cell activation [21,22].

In this study, we aimed to test whether NFL, HoxB3, and YKL-40 could potentially be used for the prediction of disease activity and clinical outcomes in NBD. For this purpose, we measured levels of these biomarkers in CSF samples obtained and stored during NBD attacks and looked for potential correlations with clinical activity parameters of NBD documented over several years of follow-up.

## 2. Materials and methods

### 2.1. Patients

Twenty-three NBD patients fulfilling the diagnostic criteria for BD [23] were enrolled. Demographical and clinical features of NBD patients are shown in Table 1. CSF samples were obtained from all NBD patients during an active episode. Typical features of parenchymal NBD (unilateral or bilateral large hyperintense lesions extending from brainstem to diencephalon and basal ganglia) and cerebral venous thrombosis (nonparenchymal NBD) were demonstrated by magnetic resonance imaging (MRI) and magnetic resonance venography when required. NBD patients with other coexisting disorders and acute infections were excluded. None of the patients were under immunosuppressive treatment during CSF sampling. NBD patients were followed thereafter under a standard treatment protocol including pulse intravenous methylprednisolone during attacks and azathioprine. The progression of disability was graded by a modified Rankin score (mRS), which was recorded during CSF sampling and the final follow-up visit. The difference between the last and first mRS scores (Δ mRS) was calculated. Duration of NBD, the total number of attacks, and the number of attacks during the follow-up period (between CSF sampling and final visit) were documented. Cognitive impairment was specifically interrogated during follow-up visits and was defined as attention deficit or memory loss that interferes with the ability to perform daily household activities (e.g., cooking, cleaning) and to work and engage in social activities outside the home. The study was approved by Institutional Review Board and informed consent was received from each participant.

### 2.2. Elisa

All CSF samples were collected during NBD attacks and stored at −80 °C until use. The levels of NFL (IBL International, Hamburg, Germany, UD51001), YKL-40 (SunRed Biotechnology Company, Shanghai, China), and HoxB3 (SunRed Biotechnology Company) were measured by ELISA according to the manufacturer’s instructions. Normal values of NFL were measured between 112 and 821 pg/mL as per the manufacturer’s instructions. Optical densities were measured at 450 nm and concentrations were calculated by reference to the standard curves.

### 2.3. Statistical analysis

A comparison of parametric data among patient subgroups was conducted by Student’s t-test. mRS and Δ mRS values were compared by Mann-Whitney U. Categorical parameters were compared with chi-square test. Correlation studies were accomplished by Spearman’s correlation test. P-value below 0.05 was defined as statistical significance.

## 3. Results

### 3.1. Comparison of parenchymal and nonparenchymal NBD patients

NBD patients (13 men and 10 women) had an average age (± standard deviation) of 40.5 ± 14.9 years and were classified as parenchymal NBD (n = 16) and nonparenchymal NBD (n = 7). The average mRS recorded during attacks (when CSF samples were obtained) was 1.5 ± 1.1 and was increased to 1.9 ± 1.5 during a follow-up duration of 3.9 ± 1.3 years (range 2–6 years). Thirteen of 23 NBD patients exhibited elevated mRS and 5 patients declared significant cognitive impairment during follow-up (Table 1). While 8 NBD patients showed reduced mRS, mRS of 2 patients remained identical throughout the follow-up period.

Parenchymal and nonparenchymal NBD patients showed comparable age, gender, disease duration, and follow-up duration. However, parenchymal NBD patients had higher initial mRS, final mRS and number of attacks in total and after CSF collection. Also, 12 of 13 NBD patients with elevated mRS and all 5 NBD patients, who developed significant cognitive impairment during follow-up, were within the parenchymal group. CSF NFL, HoxB3, and YKL-40 levels of parenchymal NBD patients were significantly higher than those of nonparenchymal patients (Table 2).

### 3.2. Comparison of NBD patients with and without stable clinical course

To better evaluate the association of CSF biomarkers with the clinical progression of NBD, patients were grouped as those having exhibited (n = 13) and not exhibited (n = 10) an increase in their mRS values during follow-up. While these two groups were matched in terms of age, gender, NBD duration, follow-up duration, and initial mRS, the elevated-mRS group showed a higher number of patients with parenchymal involvement and cognitive impairment and significantly elevated final mRS values and attack numbers. Moreover, patients with increased mRS displayed significantly elevated CSF NFL levels than patients with a relatively stable disease course. By contrast, CSF levels of HoxB3 and YKL-40 were comparable among mRS subgroups (Table 3). The elevated-mRS group had higher parenchymal NBD prevalence and a relatively higher follow-up duration. When only parenchymal NBD patients with and without mRS elevation were compared, the NFL level difference between mRS subgroups ceased to be significant (Table 4), suggesting that increased NFL in the elevated mRS group was a reflection of higher parenchymal NBD prevalence and follow-up duration in this subgroup.

### 3.3. Comparison of NBD patients with higher and lower CSF NFL levels

Since CSF HoxB3 and YKL-40 levels did not show any association with clinical worsening, we focused on CSF NFL levels for the remainder of our study. To have a better insight into the association between CSF NFL levels and clinical progression of NBD, patients with higher and lower CSF NFL were compared. While patients with higher and lower NFL levels showed comparable age, gender, NBD duration, follow-up duration, the total number of attacks, initial and final mRS scores, NBD patients with CSF NFL levels of ≥1000 ng/L were more likely to have parenchymal involvement, new attacks and cognitive impairment during follow-up. As a matter of fact, NBD patients with CSF NFL levels <1000 ng/L did not exhibit any NBD attacks or symptoms of cognitive impairment in a follow-up duration of 3.6 ± 1.2 years. CSF levels of HoxB3 and YKL-40 were comparable among NBD patients with high and low NFL levels (Table 5). When only parenchymal NBD patients with high and low NFL levels were compared, similar tendencies were observed. Despite having a relatively younger age and reduced disease duration, parenchymal NBD patients with a CSF NFL level of more than 1000 ng/L were more likely to develop cognitive impairment and new attacks during follow-up (Table 6).

Next, we investigated potential correlations between CSF NFL levels and clinical, demographic, and CSF biomarker variables listed in Table 1. CSF NFL levels were only correlated with disease duration (p = 0.009, R = 0.612) and mRS at the last visit (p = 0.049, R = 0.400). While, CSF HoxB3 levels were correlated with a number of attacks during the follow-up (p = 0.010, R = 0.539), CSF YKL-40 levels were not correlated with any of the parametric variables. There were no correlations between CSF biomarker levels versus age, disease duration, total number of attacks, initial mRS and Δ mRS. There were also no correlations among CSF levels of NFL, HoxB3, and YKL-40.

## 4. Discussion

In this study, we measured CSF levels of NFL, HoxB3, and YKL-40 in NBD patients for the first time, to our knowledge. We preferred CSF over serum measurements since CSF levels of biomarkers are expected to be more closely related to disease activity in inflammatory and degenerative disorders. Moreover, we recently showed that CSF HoxB3 and YKL-40 levels had a higher predictive value than serum HoxB3 and YKL-40 levels in the evaluation of disability progression in MS patients [20]. Several disease activity parameters including mRS value, mRS elevation, the number of attacks, and cognitive impairment were significantly increased in parenchymal patients as compared to vascular NBD patients with similar clinical and demographic features, as described previously [4,5]. Likewise, parenchymal NBD patients showed higher levels of all three biomarker candidates than nonparenchymal patients. Higher CSF NFL, HoxB3, and YKL-40 levels in parenchymal NBD may plausibly be a reflection of elevated neuroaxonal degeneration, immune system activation, and glial activation, respectively, in this NBD subgroup.

An intriguing feature was that CSF YKL-40 levels were highly elevated (>900 pg/mL) in all NBD patients regardless of disease activity. This is in contrast with MS patients of our outpatient clinic, who display CSF YKL-40 levels in an approximate range of 40–1200 pg/mL and an average of around 700 pg/mL [20]. In the CNS, YKL-40 is mainly produced by activated infiltrating macrophages, neutrophils and glial cells [24]. Critical molecular pathways of innate immunity (e.g., inflammasome complex) are significantly enhanced in NBD, particularly during clinical attacks [25,26]. Therefore, homogeneously elevated YKL-40 levels may be caused by highly increased glial activity and infiltrating macrophages/neutrophils, which are hallmarks of NBD pathology [2,3].

In MS, increased CSF NFL levels predict enhanced disability in the upcoming years. Higher CSF NFL is usually linked to more advanced disability and a greater likelihood of cognitive dysfunction in MS [27]. Our results suggest that CSF NFL may have similar power in the prediction of forthcoming somatic and cognitive disability in NBD. In both the total and parenchymal NBD groups, higher NFL levels measured in the attack CSF corresponded to an increased number of attacks, increased disability, and occurrence of cognitive symptoms during follow-up. More specifically, CSF NFL levels >1000 ng/L were associated with the occurrence of new attacks and the emergence of significant cognitive dysfunction in the forthcoming ~3–4 years. By contrast, patients with CSF NFL levels of less than 1000 ng/L did not display any attacks or cognitive dysfunction during the same time period. HoxB3 levels were only marginally correlated with attack numbers and YKL-40 levels were not associated with any of the clinical progression parameters decreasing the enthusiasm for further investigation of these biomarkers in NBD.

Interestingly, there was no correlation between CSF NFL levels versus mRS values and attack numbers documented during the NBD attack. This finding suggests that cross-sectional NFL level is not a good indicator of NBD-related disability accumulated in the past. This is the opposite of the MS experience, which suggests that CSF NFL is correlated with both cross-sectional and longitudinal and prospective disability in MS patients [28].

It was also notable that NFL level was only marginally correlated with an increase in mRS values during follow-up, despite showing a stronger association with attack numbers and cognitive dysfunction. This weak association with mRS, mostly an indicator of somatic disability, might be because this scoring system is not an ideal method for measuring disability in BD/NBD. Since BD is a systemic disease, patients may show physical impairment due to nonneurological or noninflammatory factors, such as severe mechanical joint problems or peripheral blood vessel occlusion [29,30]. Thus, the absence of an NBD-specific disability scale is a major obstacle in prognostic biomarker studies. Secondly, cognitive impairment and brain atrophy might be developing in NBD on a steady-state basis independently of neurological attacks, increase in scales of somatic disability, or presence of MRI lesions [31,32]. Moreover, brain volume loss may be observed in NBD in brain regions (e.g., hippocampus) that are not among typical lesion sites of NBD [31]. As demonstrated in MS patients, continuously enhanced glial activity might be one of the driving forces behind this concealed progression of cognitive disability, since enhanced microglial activity does not manifest in the form of clinical exacerbations and MRI lesions [33]. Proteolytic enzymes and neurotoxic cytokines produced by M1 microglia may lead to chronic and insidious neuronal loss leading to cognitive dysfunction in due time [34]. This possibility prompts the requirement for nonradiological and molecular-based prognostic biomarkers such as NFL.

In our study, diagnosis of cognitive dysfunction was based on patients’ statements and complaints and thus we could only pinpoint patients with the most severe cognitive problems. Thus, a major limitation of our study was the absence of formal cognitive tests that would more accurately diagnose cognitive dysfunction and also single outpatients with milder cognitive impairment. Likewise, the absence of imaging data obtained during the final follow-up visit prevented assessment of the association between NFL levels and brain atrophy.

In brief, our results suggest that CSF NFL levels higher than 1000 ng/L might indicate an unfavorable prognosis in NBD and thus these patients should be more closely monitored and alternative treatment options such as cyclophosphamide should be considered. These results should be further validated by higher numbers of patients with longer follow-up durations.

## Figures and Tables

**Table 1:** Clinical, demographic features and cerebrospinal fluid (CSF) measurements of neuro-Behçet’s disease (NBD) patients.

**Age**	40.5 ± 14.9
**Gender (men/women)**	13/10
**Parenchymal/non-parenchymal NBD**	16/7
**Duration of NBD (years)**	11.7 ± 7.8
**Follow-up after CSF collection (years)**	3.9 ± 1.3
**First mRS during CSF collection**	1.5 ± 1.1
**Final mRS**	1.9 ± 1.5
**Elevation of mRS during follow-up**	13 patients
**Total number of attacks**	1.5 ± 0.7
**Number of attacks during follow-up**	0.3 ± 0.6
**Patients with cognitive symptoms**	5
**CSF level of NFL (ng/L)**	3401.6 ± 3876.5
**CSF level of HoxB3 (ng/L)**	3.3 ± 0.7
**CSF level of YKL-40 (pg/mL)**	1096.0 ± 91.6

Numerical data are denoted as mean ± standard deviation.

mRS: modified Rankin score; NFL: neurofilament light chain; HoxB3: homeobox protein Hox-B3.

**Table 2:** Comparison of clinical, demographic features, and cerebrospinal fluid (CSF) measurements of neuro-Behçet’s disease (NBD) patients with and without parenchymal involvement.

	Parenchymal NBD (n = 16)	Nonparenchymal NBD (n = 7)	p-value
**Age**	42.3 ± 12.0	36.4 ± 20.5	0.250
**Gender (men/women)**	9/7	4/3	0.968
**Duration of NBD (years)**	12.0 ± 6.9	11.0 ± 10.3	0.411
**Follow-up after CSF collection (years)**	4.3 ± 1.4	3.0 ± 0.6	0.115
**First mRS during CSF collection**	1.8 ± 1.2	0.9 ± 0.7	**0.012**
**Final mRS**	2.6 ± 1.2	0.3 ± 0.8	**<0.001**
**Elevation of mRS during follow-up**	12 patients	1 patient	**0.025**
**Total number of attacks**	1.7 ± 0.8	1.0 ± 0.0	**0.002**
**Number of attacks during follow-up**	0.5 ± 0.6	0.0 ± 0.0	**0.007**
**Patients with cognitive symptoms**	5	0	0.095
**CSF level of NFL (ng/L)**	4583.9 ± 4125.8	699.1 ± 543.3	**0.001**
**CSF level of HoxB3 (ng/L)**	3.5 ± 0.8	3.0 ± 0.2	**0.015**
**CSF level of YKL-40 (pg/mL)**	1122.8 ± 97.9	1034.7 ± 22.2	**0.002**

Numerical data are denoted as mean ± standard deviation.

mRS: modified Rankin score; NFL: neurofilament light chain; HoxB3: homeobox protein Hox-B3.

Bold p values indicate statistical significance.

**Table 3:** Comparison of clinical, demographic features, and cerebrospinal fluid (CSF) measurements of neuro-Behçet’s disease (NBD) patients with and without elevated mRS during follow-up.

	NBD with stable mRS (n = 10)	NBD with elevated mRS (n = 13)	p-value
**Age**	36.5 ± 15.6	43.6 ± 14.1	0.137
**Gender (men/women)**	6/4	7/6	0.768
**Parenchymal/nonparenchymal NBD**	4/6	12/1	**0.007**
**Duration of NBD (years)**	11.5 ± 9.9	11.8 ± 6.2	0.457
**Follow-up after CSF collection (years)**	3.6 ± 1.3	4.1 ± 1.4	0.514
**First mRS during CSF collection**	1.6 ± 1.2	1.5 ± 1.1	0.389
**Final mRS**	0.6 ± 1.1	2.9 ± 1.0	**<0.001**
**Total number of attacks**	1.1 ± 0.3	1.8 ± 0.8	**0.007**
**Number of attacks during follow-up**	0.0 ± 0.0	0.6 ± 0.7	**0.006**
**Patients with cognitive symptoms**	1	4	0.231
**CSF level of NFL (ng/L)**	1848.1 ± 3022.0	4596.5 ± 4140.3	**0.040**
**CSF level of HoxB3 (ng/L)**	3.1 ± 0.4	3.5 ± 0.8	0.081
**CSF level of YKL-40 (pg/mL)**	1096.9 ± 131.7	1095.2 ± 48.6	0.485

Numerical data are denoted as mean ± standard deviation.

mRS: modified Rankin score; NFL: neurofilament light chain; HoxB3: homeobox protein Hox-B3.

Bold p values indicate statistical significance.

**Table 4:** Comparison of clinical, demographic features, and cerebrospinal fluid (CSF) measurements of parenchymal neuro-Behçet’s disease (NBD) patients with and without elevated mRS during follow-up.

	NBD with stable mRS (n = 4)	NBD with elevated mRS (n = 12)	p value
**Age**	45.6 ± 14.0	41.2 ± 11.8	0.302
**Gender (men/women)**	3/1	6/6	0.383
**Duration of NBD (years)**	13.2 ± 9.1	11.6 ± 6.4	0.380
**Follow-up after CSF collection (years)**	4.3 ± 1.9	4.3 ± 1.4	0.960
**First mRS during CSF collection**	2.5 ± 1.3	1.6 ± 1.1	0.132
**Final mRS**	1.5 ± 1.3	3.0 ± 1.0	**0.048**
**Total number of attacks**	1.3 ± 0.5	1.9 ± 0.8	**0.047**
**Number of attacks during follow-up**	0.0 ± 0.0	0.6 ± 0.7	**0.005**
**Patients with cognitive symptoms**	1	4	0.755
**CSF level of NFL (ng/L)**	3513.8 ± 4545.6	4940.6 ± 4125.7	0.302
**CSF level of HoxB3 (ng/L)**	3.2 ± 0.7	3.5 ± 0.8	0.246
**CSF level of YKL-40 (pg/mL)**	1197.3 ± 170.3	1097.9 ± 49.7	0.165

Numerical data are denoted as mean ± standard deviation.

mRS: modified Rankin score; NFL: neurofilament light chain; HoxB3: homeobox protein Hox-B3.

Bold p values indicate statistical significance.

**Table 5:** Comparison of clinical, demographic features, and cerebrospinal fluid (CSF) measurements of neuro-Behçet’s disease (NBD) patients with higher (<1000 ng/L) and lower (≥1000 ng/L) CSF NFL levels.

	NBD with lower NFL (n = 10)	NBD with higher NFL (n = 13)	p-value
**Age**	44.6 ± 20.4	37.4 ± 8.2	0.155
**Gender (men/women)**	6/4	7/6	0.768
**Parenchymal/nonparenchymal NBD**	5/5	11/2	0.074
**Duration of NBD (years)**	14.5 ± 7.9	9.5 ± 7.3	0.068
**Follow-up after CSF collection (years)**	3.6 ± 1.2	4.1 ± 1.4	0.521
**First mRS during CSF collection**	1.5 ± 1.3	1.5 ± 1.1	0.470
**Final mRS**	1.5 ± 1.6	2.2 ± 1.5	0.136
**Elevation of mRS during follow-up**	4 patients	9 patients	0.161
**Total number of attacks**	1.4 ± 0.7	1.6 ± 0.8	0.286
**Number of attacks during follow-up**	0.0 ± 0.0	0.5 ± 0.7	**0.013**
**Patients with cognitive symptoms**	0	5	**0.026**
**CSF level of NFL (ng/L)**	545.6 ± 332.2	5598.5 ± 3929.5	**<0.001**
**CSF level of HoxB3 (ng/L)**	3.3 ± 0.7	3.3 ± 0.7	0.421
**CSF level of YKL-40 (pg/mL)**	1108.9 ± 131.4	1086.0 ± 46.6	0.305

Numerical data are denoted as mean ± standard deviation.

mRS: modified Rankin score; NFL: neurofilament light chain; HoxB3: homeobox protein Hox-B3.

Bold p values indicate statistical significance.

**Table 6:** Comparison of clinical, demographic features, and cerebrospinal fluid (CSF) measurements of parenchymal neuro-Behçet’s disease (NBD) patients with higher (<1000 ng/L) and lower (≥1000 ng/L) CSF NFL levels.

	NBD with lower NFL (n = 5)	NBD with higher NFL (n = 11)	p-value
**Age**	50.7 ± 15.4	38.5 ± 8.4	0.079
**Gender (men/women)**	3/2	6/5	0.838
**Duration of NBD (years)**	14.3 ± 6.7	10.9 ± 7.0	0.194
**Follow-up after CSF collection (years)**	4.5 ± 1.2	4.2 ± 1.6	0.776
**First mRS during CSF collection**	2.2 ± 1.3	1.6 ± 1.1	0.216
**Final mRS**	2.6 ± 1.3	2.6 ± 1.2	0.480
**Elevation of mRS during follow-up**	3 patients	9 patients	0.350
**Total number of attacks**	1.6 ± 0.9	1.7 ± 0.8	0.420
**Number of attacks during follow-up**	0.0 ± 0.0	0.5 ± 0.7	**0.027**
**Patients with cognitive symptoms**	0	5	0.069
**CSF level of NFL (ng/L)**	690.8 ± 388.5	6353.5 ± 3801.4	**<0.001**
**CSF level of HoxB3 (ng/L)**	3.6 ± 0.9	3.4 ± 0.7	0.335
**CSF level of YKL-40 (pg/mL)**	1180.6 ± 160.6	1096.5 ± 40.3	0.155

Numerical data are denoted as mean ± standard deviation.

mRS: modified Rankin score; NFL: neurofilament light chain; HoxB3: homeobox protein Hox-B3.

Bold p values indicate statistical significance.
